# Evaluation of the impact of the ARC program on national nursing and midwifery regulations, leadership, and organizational capacity in East, Central, and Southern Africa

**DOI:** 10.1186/s12913-018-3233-4

**Published:** 2018-06-04

**Authors:** Jessica M. Gross, Carey F. McCarthy, Andre R. Verani, Jill Iliffe, Maureen A. Kelley, Kenneth W. Hepburn, Melinda K. Higgins, Alphonce T. Kalula, Agnes N. Waudo, Patricia L. Riley

**Affiliations:** 1 Division of Global HIV and TB at the U.S. Centers for Disease Control and Prevention, Atlanta, USA; 2 Independent Health Systems and Nursing Workforce Consultant, Geneva, Switzerland; 3 Division of Global HIV and TB of the U.S. Centers for Disease Control and Prevention, Atlanta, USA; 4 Commonwealth Nurses and Midwives Federation, London, UK; 5 ARC and Professor Emeritus at Emory University’s Nell Hodgson Woodruff School of Nursing, Atlanta, USA; 6 ARC and a Professor at Emory University’s Nell Hodgson Woodruff School of Nursing, Atlanta, USA; 7 Emory University’s Nell Hodgson Woodruff School of Nursing, Atlanta, USA; 8 East Central and Southern Africa Health Community (ECSA-HC), Arusha, Tanzania; 9 Africa Health Workforce Project and ARC Secretariat, Nairobi, Kenya; 10 Health Systems Program Integration Team in the International Lab Branch at the U.S. Centers for Disease Control and Prevention, Atlanta, USA

**Keywords:** Nursing, Midwifery, Regulation, HIV, Capacity building, Sub-Saharan Africa, African health professions regulatory collaborative

## Abstract

**Background:**

The African Health Professions Regulatory Collaborative (ARC) was launched in 2011 to support countries in East, Central, and Southern Africa to safely and sustainably expand HIV service delivery by nurses and midwives. While the World Health Organization recommended nurse initiated and managed antiretroviral therapy, many countries in this region had not updated their national regulations to ensure nurses and midwives were authorized and trained to provide essential HIV services. For four years, ARC awarded annual grants, convened regional meetings, and provided technical assistance to country teams of nursing and midwifery leaders to improve national regulations related to safe HIV service delivery. We examined the impact of the program on national regulations and the leadership and organizational capacity of country teams.

**Methods:**

Data was collected to quantify the level of participation in ARC by each country (number of grants received, number of regional meetings attended, and amount of technical assistance received). The level of participation was analyzed according to two primary outcome measures: 1) changes in national regulations and 2) improvements in leadership and organizational capacity of country teams. Changes in national regulations were defined as advancement of one “stage” on a capability maturity model; nursing and midwifery leadership and organizational capacity was measured by a group survey at the end of the program.

**Results:**

Seventeen countries participated in ARC between 2012 and 2016. Thirty-three grants were awarded; the majority addressed continuing professional development (20; 61%) and scopes of practice (6; 18%). Fourteen countries (representing approximately two-thirds of grants) progressed at least one stage on the capability maturity model. There were significant increases in all five domains of leadership and organizational capacity (*p* < 0.01). The number of grants (Kendall’s tau = 0.56, *p* = 0.02), duration of technical assistance (Kendall’s tau = 0.50, *p* = 0.03), and number of learning sessions attended (Kendall’s tau = 0.46, *p* = 0.04) were significantly associated with improvements in in-country collaboration between nursing and midwifery organizations.

**Conclusions:**

The ARC program improved national nursing regulations in participating countries and increased reported leadership, organizational capacity, and collaboration among national nursing and midwifery organizations. These changes help ensure national policies and professional regulations underpin nurse initiated and managed treatment for people living with HIV.

**Electronic supplementary material:**

The online version of this article (10.1186/s12913-018-3233-4) contains supplementary material, which is available to authorized users.

## Background

In sub-Saharan Africa, an estimated seven million people living with HIV still need antiretroviral treatment (ART) if targets set by the United Nations Joint Program on HIV and AIDS are to be reached [1–3]. Studies from numerous countries in sub-Saharan Africa have demonstrated that nurses and midwives can provide safe and effective HIV care and treatment [4–12]. The World Health Organization (WHO) recommends nurse initiated and managed ART (NIMART) to increase ART coverage [13]. However, it is incumbent upon countries relying on NIMART to ensure that health policies authorize NIMART and national nursing and midwifery regulatory frameworks support nurses and midwives who are providing advanced HIV services [14–16].

In the region of East, Central and Southern Africa (ECSA), most countries have a nursing and midwifery regulatory council, including Mozambique as of 2016. However, the extent of regulations that support safe practices and adequate education for nurses and midwives providing HIV services varies widely across this region [17]. In a few countries, licensure and re-licensure regulations require proof of competency in HIV service delivery or continuing professional development (CPD) with HIV-related content [18, 19]. Nursing and midwifery scopes of practice in the region do not uniformly include NIMART or other task sharing practices [17, 20]. Pre-service education does not consistently prepare nurses and midwives to deliver essential HIV services by providing HIV content in the curriculum [21–23]. Lastly, policy makers and regulators confront capacity and resource constraints to implementing key regulatory reforms needed to strengthen nurse-led HIV service delivery [24].

The African Health Professions Regulatory Collaborative Program (ARC) was designed to promote the strengthening of legal and regulatory frameworks for improved HIV care and prevention [25]. The program was launched in 2011 by the United States President’s Emergency Plan for AIDS Relief (PEPFAR) and the U.S. Centers for Disease Control and Prevention (CDC) with national leaders in nursing and midwifery from 14 ECSA countries. CDC funded Emory University to implement ARC in collaboration with the East, Central and Southern Africa Health Community’s College of Nursing (ECSACON) and the Commonwealth Nurses and Midwives Federation. The goal of enhancing HIV service provision by safely expanding NIMART was supported by program objectives, including to update and improve regulations (e.g. scope of practice, CPD) that impacted the extent and quality of HIV services provided by nurses and midwives [25]. Another objective was to strengthen the leadership, collaboration, and capacity among the national nursing organizations in participating countries [26]. The ARC program consisted of grants awarded to teams of nursing and midwifery leaders from each country to focus on improving their national regulations. The country teams were supported by regular regional meetings and technical assistance. This study evaluated whether the ARC program approach was effective in improving national nursing and midwifery regulations and in increasing the capacity of and leadership by the participating nursing and midwifery organizations.

## Methods

### Design

This study used a retrospective analysis of routinely collected program data as well as post-program survey data with 17 countries. The protocol for the ARC impact evaluation was reviewed and approved by the Emory University institutional review board and the CDC Associate Director for Science. All participants gave individual voluntary informed consent.

### Participants

ARC was implemented in 14 ECSA countries for four years: Botswana, Lesotho, Kenya, Malawi, Mauritius, Mozambique, Namibia, the Seychelles, South Africa, Swaziland, Tanzania, Uganda, Zambia, and Zimbabwe. Three additional countries, Ethiopia, Rwanda, and South Sudan, participated for three years. In each country, ARC supported a four-person country team – or a Quad – comprised of a nursing or midwifery leader from the ministry of health, the national nursing and midwifery council, the professional nursing and midwives association or union, and an academic institution.

### ARC program procedures

#### Grants

Through ARC, the Quads undertook one-year grant-funded projects aimed at strengthening professional regulation and facilitating NIMART in their country [26]. Eligible topics for grants included nursing and midwifery legislation, registration systems, professional licensure, scope of practice, CPD, pre-service education accreditation, or professional conduct and discipline. Between five and 11 countries received a grant each year; grants were typically 10,000 U.S. dollars and always provided directly to the nursing and midwifery leadership teams [27]. The process for awarding grants was competitive and has been described elsewhere [28]. In some cases, country teams garnered additional financial or in-kind support for their grant project from sources such as their national government or local non-governmental organizations [29, 30].

#### Regional meetings

Two types of meetings were held for ARC country teams each year: regional meetings called “Learning Sessions” were held twice a year for countries implementing a grant that year; an annual meeting or “Summative Congress” was attended by all ARC countries. At Learning Sessions, Quads formally presented their funded projects and identified challenges and solutions they encountered during implementation. They received feedback and suggestions from their colleagues and technical consultations from ARC faculty and subject matter experts. All 17 country teams convened annually for the Summative Congress. Countries that had implemented a grant project that year would present their project to all the other Quads. International and regional experts in regulation and NIMART presented on topics related to the countries’ grant topics. Networking events were held at each meeting and helped deepen relationships among and across country teams and encourage cross-country collaboration on common regulatory issues.

#### Technical assistance

Technical assistance (TA) was provided to country teams implementing a grant. Most often, a member of the ARC faculty or a subject matter expert visited the Quad in-country; occasionally the TA was provided remotely. In certain circumstances, Quads that did not receive a grant could still get TA on the topic they identified in their (unfunded) grant proposal. These Quads would attend a Learning Session that year to receive in-person TA from the ARC faculty and experts present, as well as benefit from the speakers and country presentations in the Learning session. This overall structure of ARC—country teams working collaboratively on an improvement project and regularly convening to assess progress—was designed to mimic the Institute for Healthcare Improvement’s model for “breakthrough change” [26, 31].

Grants, regional meetings, and TA were reinforced by a virtual community of practice using the Knowledge Gateway^1^ platform. Through the Knowledge Gateway, ARC offered policy and regulation resources, such as the ARC CPD Toolkit [32] and the ARC Legal Regulatory Matrix [33]. The Quads shared regulatory tools (e.g. a “needs assessment” for CPD) and documents (e.g. national scopes of practice) with each other and took advantage of the online discussion board to communicate about their experiences tackling similar regulatory challenges related to advancing NIMART [28].

### Variables

Variables used in this evaluation include ARC program data (grants, regional meetings, TA) and two primary outcomes: 1) changes in national regulations and 2) improvements in leadership and organizational capacity.

1) Program Data

- Grants: the number of grants a country team received and the topic (e.g. SOP, CPD, nursing legislation)
- Regional Meetings: the number of Learning Sessions and Summative Congresses attended by the Quad
- Technical Assistance: the amount and type (in-country, remote, learning session if non-grantee) of TA received by country teams. Three levels were developed to quantify TA; the levels for all four years were added together for a composite TA score, which could range from 0 to 12:
   ○ Level 1: remote TA only (e.g. phone or email consultations, document review)
   ○ Level 2: one in-country or in-person consultation by an ARC TA provider, with or without additional remote TA
   ○ Level 3: two or more in-country or in-person consultations by an ARC TA provider

2) National Nursing and Midwifery Regulations: In order to assess if regulations changed over the course of the ARC program, each country reported on the status of seven nursing and midwifery regulations each year. In addition, country teams that received a grant would provide a “pre” and “post” status on the regulation addressed by their grant that year. Changes in regulations were measured using the Regulatory Function Framework (RFF), described below.

3) Leadership and Organizational Capacity: Country teams were asked to report on five specific domains of leadership, collaboration, and organizational capacity. The domains were selected and defined relative to the initial objectives of the ARC program [26]:

1. Teamwork: the national nursing and midwifery leaders (“the Quad”) work together effectively as a team
2. In-country Collaboration: the national nursing and midwifery organizations collaborate with each other to accomplish mutual goals
3. Intra-professional Collaboration: the national nursing and midwifery organizations collaborate with other in-country organizations
4. Regional Collaboration: the national nursing and midwifery leaders network and collaborate with nursing and midwifery leaders from other countries
5. Resource Mobilization: the national nursing and midwifery leaders garner resources and funding, beyond that provided by ARC, to advance nursing and midwifery regulation within their countries.

### Instruments

1) Data on the major components of the ARC program (i.e. grants, regional meetings, TA) were collected via routine program management by Emory University and used for reporting to CDC and PEPFAR.

2) Changes in regulatory functions were measured by the Regulatory Function Framework (RFF). The creation of the RFF was led by the CDC to measure the impact of the ARC program on national regulations. The RFF comprises seven regulatory functions, including nursing and midwifery legislation, registration systems, licensure process, SOP, CPD, pre-service education accreditation, and professional conduct and discipline [34]. The RFF allows for assessment of the regulatory functions in terms of five distinct stages: “ad hoc” (stage 1), “documented” (stage 2), “routine” (stage 3), “improved” (stage 4), and “optimized” (stage 5) [34]. The stages are sequential and each stage is characterized by elements of regulation that must be in place; advancing a stage represents a meaningful step towards a more optimal regulation [35]. A description of the stages for each regulatory function and their respective elements are presented in Additional file 1. The development of the RFF and results of use by the ARC program has been published previously [27, 34, 36].

3) Changes in leadership and organization capacity were assessed by a survey instrument designed by the ARC faculty. The survey included qualitative and quantitative Likert scale questions, which asked country teams to rate themselves in the five domains of leadership, collaboration, and organizational capacity both before and after their participation in the ARC program. A detailed description of the survey’s qualitative results has been published elsewhere [37].

### Data collection

1) Program data were collected annually at the Summative Congress and throughout year as TA occurred.

2) At the Summative Congress, Quads discussed the stages of each of the seven regulatory functions and provided the information to ARC faculty. These data were collected as an annual “point in time” assessment of in all seven regulatory functions on the RFF for each country. For countries that received a grant that year, “before grant” and “after grant” data were collected about the targeted regulation. Data collection on regulations took place at the ARC Summative Congresses in June 2012, June 2013, February 2015, and February 2016.

3) Country teams completed one survey per country to facilitate discussion and agreement regarding their leadership, collaboration, and organizational capacity before and after their participation in the ARC initiative. The survey was administered at the ARC Summative Congress in February 2016.

### Analysis

We tabulated the program data on grants, regional meetings and TA from all four years. The two primary program outcomes (1) changes in regulations and (2) improvements in leadership and organizational capacity were analyzed according to their relationship to the program data. We compared the regulation data collected from the RFF in June 2012 (Year 1) to the data collected in February 2016 (Year 4) for all 17 countries; improvement was defined as movement of at least one stage on the RFF. Relevant country-level characteristics, including the number of years in ARC, English as a national language, a country’s regional location, and receipt of PEPFAR support, were included in the analysis. Analysis for both outcomes included checking the ordinal and categorical data for completeness and reviewing for accuracy. Non-parametric statistical tests (i.e. Mann-Whitney U tests, Wilcoxon rank sum paired tests, and Kendall’s tau correlations), as well as chi-square tests were performed to test for significant changes on the RFF from year 1 to year 4 and to assess associations between ARC program data, the two primary outcomes, and relevant country-level characteristics. SPSS v.23® was used for statistical analysis with 5% level of significance for all hypothesis tests.

## Results

### ARC program: Grants, meetings, technical assistance

#### Grants

Between 2012 and 2016, ARC awarded a total of 33 competitive grants addressing specific regulatory functions to improve HIV care provided by nurses and midwives; all 17 countries received at least one grant (Table 1). The majority of grants (61%) supported the establishment and strengthening of national CPD programs and linking licensure renewal to specific CPD requirements, such as content on HIV and AIDS care. Six grants (18%) supported nursing and midwifery SOP projects, followed by three grants (9%) to strengthen the licensure process. Two grants (6%) focused on establishing or revising nursing and midwifery legislation. Lastly, one grant (3%) focused on pre-service nursing and midwifery program accreditation and one (3%) on enhancing the nursing and midwifery registration system. Four countries implemented grants addressing two regulatory topics.

**Table 1:** Table of ARC grantees by Country, number of Grants, regulatory function and advancement

Country	# ARC grants received	Primary regulatory function (Secondary)	# Stages advanced on RFF	Highest RFF stage reached	Resulting regulatory product	Impact on HIV Care
Botswana	2	CPD	1	Stage 2	CPD Framework requiring HIV content; Scope of Practice including HIV tasks; CPD requirement for re-licensure	5 HIV-related CPD points are required; Task sharing included; CPD needed to re-license
	1	SOP	2	Stage 5		
Ethiopia	1	CPD	1	Stage 2	CPD Manual, including HIV content	Quality improvement
Kenya	1	Registration	0	Stage 4	Establishment of decentralized council offices; Pediatric HIV CPD module;	Convenient re-licensing; HIV CPD to recertify
	1	CPD	0	Stage 3		
Lesotho	3	CPD (Licensure)	3	Stage 5	Trainings to increase CPD compliance; CPD requirement for re-licensure and CPD provider accreditation application; CPD Framework and Logbook	Increased CPD compliance; 3 of 12 CPD points are required in HIV-related CPD; Instituted CPD for nurses
Malawi	1	CPD	1	Stage 4	Trainings to implement CPD mandate	Increased CPD compliance
Mauritius	1	Legislation	1	Stage 2	Regulation of Profile for Educators	Quality nursing education
Mozambique	2	Licensure (Accreditation)	0	Stage 1	Objective Structured Clinical Exam (OSCE) assessment for new nursing graduates; Law establishing Nursing Council	Assessment of PMTCT competencies for new nurses; Professional regulation
	0 (TA only)	Legislation	2	Stage 3		
Namibia	1	CPD	1	Stage 3	Survey assessed CPD compliance factors	Data to drive compliance
Rwanda	1	SOP	1	Stage 3	Scope of Practice, including HIV tasks; CPD module on HIV services	Task sharing included; HIV CPD available
	1	CPD	1	Stage 2		
Seychelles	1	Legislation	4	Stage 5	Proposed amendments to Nursing Act; Scope of Practice for HIV Generalists and Specialists; CPD Framework including HIV content	Clear HIV practice scope; CPD available to nurses; Improve education/practice
	1	SOP	0	Stage 2		
	1	CPD	1	Stage 2		
South Africa	1	CPD (Accreditation)	1	Stage 2	HIV Specialization for nurses; CPD accreditation for nurse led ART	Incentive for task sharing; Quality ART task sharing
	1	Accreditation	0	Stage 3		
South Sudan	1	SOP	0	Stage 1	Scope of Practice, including HIV tasks	Clear HIV practice scope
Swaziland	2	CPD	2	Stage 4	CPD Framework, including HIV content; Entry to practice license exam includes HIV	HIV CPD required to relicense; HIV competencies required
	1	Licensure	2	Stage 4		
Tanzania	2	CPD	1	Stage 2	CPD Framework, including HIV content; Task Sharing Policy, includes HIV services	HIV CPD required to relicense; Increased HIV task sharing
Uganda	2	SOP	2	Stage 3	Scope of Practice for Nurses and Midwives, Uganda Nursing Council, 2015	Nurses are authorized to initiate patients on ART
Zambia	2	CPD (Licensure)	1	Stage 2	CPD requirement for licensure renewal; Nurse led ART accreditation guidelines	HIV CPD required to relicense; Quality ART task sharing
Zimbabwe	2	CPD	2	Stage 5	NIMART Mentorship Training Program; CPD requirement for licensure renewal	Quality ART task sharing; CPD required to relicense

#### Regional meetings

ARC convened 13 collaborative regional meetings (i.e. Learning Sessions and Summative Congresses) in nine African locations over the course of the initiative (Additional file 2). Five countries (29.4%) attended 9–10 collaborative regional meetings over the four years (Botswana, Lesotho, Seychelles, Swaziland, and Zambia). Seven countries (51.2%) attended 7–8 collaborative regional meetings (Kenya, Mozambique, Rwanda, South Africa, Tanzania, Uganda, and Zimbabwe). Five countries (29.4%) attended 5–6 collaborative regional meetings (Namibia, South Sudan, Ethiopia, Malawi, and Mauritius). There was a relationship between the number of grants received and the number of collaborative regional meetings attended (Kendall’s tau = 0.91, *p* < 0.01). The number of learning sessions was also directly correlated with the number of years a country participated in ARC (Kendall’s tau = 0.53, *p* < 0.02); however, the number of years in ARC was not significantly associated with the number of grants a country received (Kendall’s Tau = 0.37, *p* = 0.12).

#### Technical assistance

**Fig. 1:**
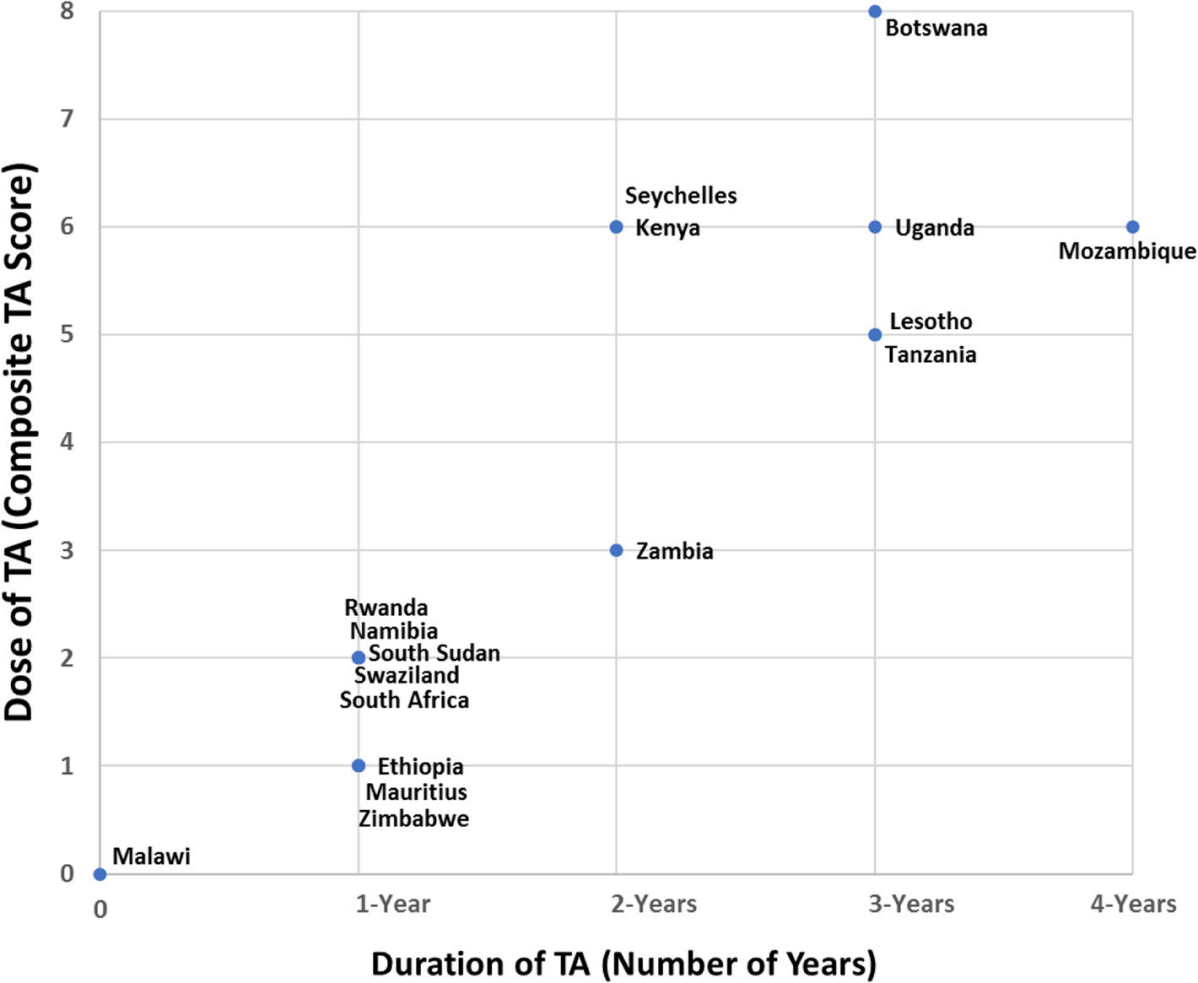
Range of Technical Assistance Received by Duration (# Years) and Dose (Composite TA Score)

The number of years of receiving TA and the composite TA scores were calculated separately for each country (Fig. 1). The years of TA ranged from zero to 4-years. One country (6%), Malawi, did not receive any TA. One country (6%), Mozambique, received the maximum of four years of TA to advance national nursing and midwifery legislation to establish a regulatory council. Fifteen countries (88%) received between one and three years of TA; the most common number of years of TA received was one (eight countries, 47%), followed by three (four countries, 24%). The composite TA scores ranged from zero (Malawi) to eight (Botswana). Seven countries (41%) had composite TA scores of five and above; while, nine countries (53%) had a composite TA scores of two or below. There was a clustering of nine countries with low duration (one year or less) of TA and low composite TA scores (two and below); this clustering was not mirrored in countries with high TA scores: seven countries (41%) with composite TA scores of five and above ranged in duration of TA between two and four years. There was a correlation between the number of grants received and the number of years of TA received (Kendall’s tau = 0.49, *p* < 0.03), as well as the composite TA score (Kendall’s tau = 0.56, *p* < 0.01).

### National Nursing and midwifery regulations

**Fig. 2:**
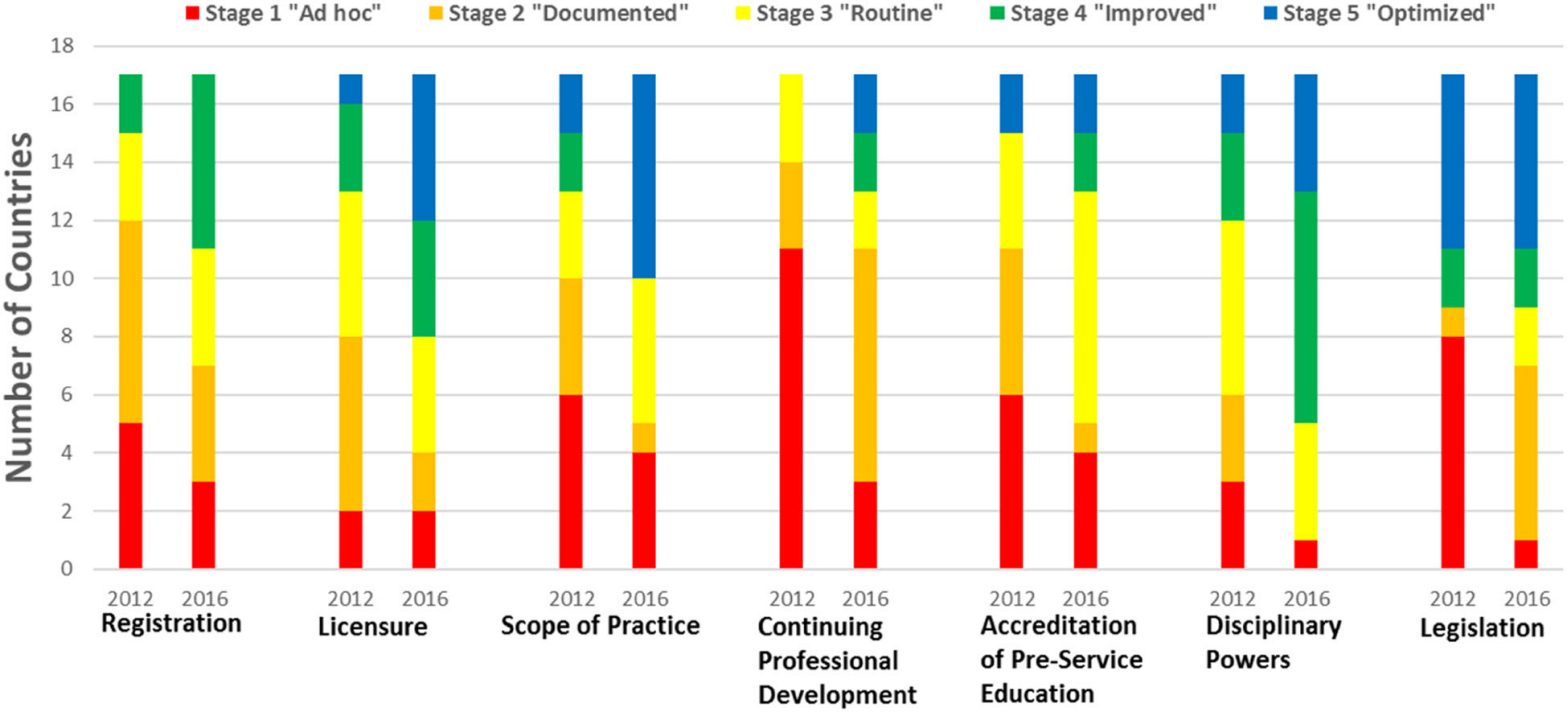
Countries’ Regulatory Function Maturity at Baseline (2012) and Endline (2016) of ARC (n = 17 countries)

Over the course of the ARC initiative (2012–2016), countries reported advancements on the RFF across all seven regulations (Fig. 2). There were significant advancements in five regulations including CPD (p < 0.01), professional discipline (*p* < 0.01), SOP (*p* = 0.01), licensure (*p* < 0.02), and registration (*p* = 0.03); advancements for accreditation (*p* < 0.06) and legislation (*p* = 0.32) were not statistically significant. In 2012, CPD was the least-developed regulation in the region: 11 of the 17 countries (65%) were in Stage 1, indicating unorganized or ad hoc approaches to CPD; by the end of ARC, only three countries (18%) were in Stage 1. The SOP function was also under-developed at the start of ARC: ten countries (59%) were in the earliest two stages of development; by the end of ARC, only five countries (29%) were in these stages and seven (41%) countries had reached to the most advanced stage.

Among countries reporting pre- and post- changes in the regulation topic addressed by their one-year grant, 14 countries (representing 20 of 33 grants) progressed at least one stage on the RFF per project grant (Table 1). We also examined the singular effect of receiving a grant upon regulatory function improvement by comparing advancement on the RFF by countries that received a grant in that function and those that did not. The 13 countries that received CPD grants (61% of grants) improved their CPD stage significantly more from year 1 to year 4 (median improvement of 1 stage) than those without a CPD grant (median improvement 0 stages). The five countries that received SOP grants also improved by a median of 1 stage; however, the median improvement in this function by countries that did not receive a grant (0.5 stages) was not statistically different. We noted no statistical difference between grantees’ and non-grantees’ median improvements for licensure, accreditation, and legislation functions. Only one country received a registration function grant, and none received a disciplinary function grant.

### Leadership and organizational capacity

**Fig. 3:**
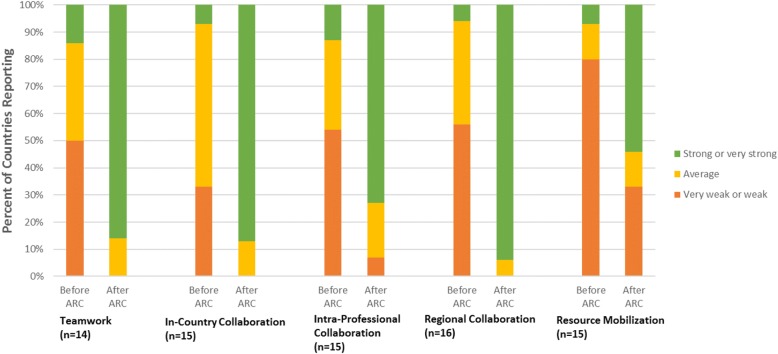
ARC’s Impact on National Nursing Leaders’ Teamwork, Collaboration and Resource Mobilization (n = number of countries)

We found significant increases in the country teams’ ranking of leadership and organizational capacity before and after the four-year ARC initiative for all five capacity domains (*p* < 0.01) (Fig. 3). While the changes in all five capacity and leadership domains were statistically significant, three had the largest effect size, including: 1) teamwork among a country’s nursing and midwifery leaders, 2) in-country collaboration among nursing/midwifery organizations, and 3) regional collaboration with nursing and midwifery leaders from other countries. Less than 15% of countries scored these domains as strong or very strong before ARC, compared to more than 85% following the initiative. For the remaining domains, less than 13% of countries scored intra-professional collaboration as strong or very strong before ARC, compared to 73% after the initiative; resource mobilization improved from 7% of countries scoring strong or very strong before ARC to 54% after the initiative. A more detailed and qualitative description of changes in the nursing and midwifery capacity domains is reported elsewhere [37].

An analysis of the program data outputs with the nursing and midwifery leadership and capacity domains identified five significant relationships. In the domain of in-country collaboration between nursing organizations, the number of grants (Kendall’s tau = 0.56, *p* = 0.02), duration of TA (Kendall’s tau =0.50, *p* = 0.03), and number of learning sessions attended (Kendall’s tau = 0.46, *p* = 0.04) were all significantly associated with improvements. The duration of TA was significantly associated with improvements in intra-professional collaboration (Kendall’s tau = 0.44, *p* = 0.05) and the ability to mobilize resources (Kendall’s tau = 0.60, *p* = 0.01).

## Discussion

We evaluated the four-year 17-country ARC initiative by assessing the major program components (grants, regional meetings, and TA) and analyzing their relationship to two outcome variables: improvements in national regulations and increases in nursing and midwifery leadership and organizational capacity. We found the ARC program to be effective in improving national regulations: most grantees (20 of 33) progressed at least one stage on the RFF regardless of regulatory function. While not all progress on the RFF was statistically significant, a gain of any stage on an instrument such as the RFF indicates meaningful improvement [35]. The greatest improvements for the region were in the regulations of CPD and SOPs. Thirteen countries that received CPD grants demonstrated statistically significant improvements in this regulatory function. Three countries made statistically significant advancements in developing or updating the nursing and midwifery SOP.

Because ARC focused “upstream” on regulations, instead of directly at point of service provision, the improvements reflect crucial and sustainable changes in national health policies. For example, seven countries (Botswana, Ethiopia, Rwanda, Seychelles, South Africa, Tanzania, and Zambia) established national CPD programs and made CPD mandatory for licensure renewal. Four additional countries (Malawi, Swaziland, Lesotho, and Zimbabwe) reported that content on HIV service delivery must be included in CPD for nurses and midwives. The improvements in SOPs in three countries (Botswana, Rwanda, and Uganda) resulted in new or expanded SOPs that now reflect national guidelines for HIV task sharing and NIMART. The popularity of the CPD and SOP functions (26 of 33 grants) indicated clear priorities in professional regulation among nursing and midwifery leaders in the ECSA region. The ARC program not only contributed to the substantial advancement of these regulations but also fostered similar approaches by countries to improving the regulations. The result is greater harmonization of regulations in the ECSA region, which helps address increasing mobility of healthcare workers [38, 39].

The ARC initiative was also effective in developing the leadership and organizational capacity of nursing and midwifery leaders in the ECSA region. While there were improvements in self-assessment of leadership and organizational capacity across all five domains from Year 1 to Year 4, the largest effect size was seen in the domains of teamwork, in-country collaboration, and regional collaboration. These three are the most directly related domains to stated ARC objectives [28] and address some of the biggest deficits in the region. All three ARC program components – competitive grants, regional meetings, and TA – significantly improved in-country collaboration. The lack of collaboration between national nursing and midwifery organizations has been documented as a key obstacle to strengthening workforce regulations [24, 38]. Furthermore, the duration of TA significantly improved nursing and midwifery organizations’ ability to mobilize additional external resources to advance national regulatory priorities. The ability to secure financing is a critical skill to allow regulatory bodies to move towards self-sufficiency in resource-constrained environments [40].

The improvements in regulation and organizational capacity have moved ARC countries closer to global targets for HIV service delivery, health systems strengthening, and health workforce development. The 2016 WHO ART guidelines recommend using task sharing and updated regulations that facilitate NIMART to treat all people living with HIV [16]. Additional guidance from WHO recommends using national laws and health professional regulations to strenghten health systems and increase population health coverage [41]. Countries in ARC are closer to achieving several global milestones outlined in WHO’s *Global strategy for human resources for health: Workforce 2030* [39]. The *Global Strategy* underscores the need for strong regulatory mechanisms and specifically mentions strengthening the capacity of professional regulatory councils and enhancing in-country collaboration among councils, professional associations, and governments [39]. Additional principles of the *Global Strategy* include ensuring the competency and facilitating the mobility of health workers. Lastly, the WHO’s *Global Strategic Directions for Nursing and Midwifery 2016–2020* urges countries to maximize the capacities of nurses and midwives through intra- and interprofessional collaborative partnerships and continuing professional development [42].

### Limitations

This evaluation has several limitations. The RFF was developed for use within the ARC initiative to assess advancements in regulatory functions. While it went through a validation process by countries within ARC, it was not externally validated by a wider audience. The RFF was not sensitive enough to document certain sub-national or HIV specific advancements in regulations. For example, the Mozambique Quad developed an objective structured clinical examination, or OSCE, as part of the competency assessment for entry to practice. While this new examination will help ensure fitness to practice in Mozambique and is an impressive accomplishment, the licensing regulation on the RFF did not capture this development. Similarly, South Africa developed accreditation standards for a specialty certification for NIMART and tuberculosis care linked to increased remuneration. While not captured by the RFF, the development of a NIMART/NIMTB specialty certification is certainly a marked achievement in South Africa, linking advanced nursing practice to HIV care. Additionally, some countries, like Kenya, made incremental advancements that did not result in stage advancements.

Although the RFF and nurse capacity tools were administered as group surveys to facilitate discussion and improve reporting accuracy, they are both subject to social desirability bias. The nursing capacity tool is subject to recall bias. Additionally, not all regulatory advancements by countries in ARC can be attributed to the ARC initiative. During the project, other global health and PEPFAR initiatives had similar objectives of enhancing HIV service delivery by nurses and midwives and may have concurrently supported efforts that enhanced regulation or nursing and midwifery organizational capacity [43]. Furthermore, other domestic inputs may have played an important role in advancing nursing and midwifery regulation within the ECSA region. The small sample size of 17 countries, as well as the small range and measurement scale (number of years 1 to 4 and stages 1 to 5) limited the statistical analysis.

The structure of the intervention package accounts for some of the relationships identified in the results. For example, the correlation between the number of grants received and the duration and dose of TA received explains the clustering of nine countries with a fairly low TA duration (≤ 1 year) and dose (≤ 2 composite TA score). For these nine countries, over 50% received only one ARC grant. The eight countries with higher TA durations and doses all received two or more grants. The correlation between the number of grants received and the number of collaborative regional meetings attended is also due to the structure of the intervention package, since the majority of participants invited to learning sessions were grantees. Given certain elements of ARC (i.e. TA, collaborative learning sessions, exchange of regulatory tools, regional networking), countries could advance regulatory functions without necessarily needing a grant. Countries without grants also made progress in advancing regulations (e.g. disciplinary powers function) to facilitate safer HIV and health service delivery. Mozambique received four years of TA to advance national nursing and midwifery legislation, improving two stages without a grant and ending in the passage of a national law to establish a nursing council.

## Conclusion

The ARC program was effective in improving national regulations and increasing leadership and organizational capacity in the ECSA region. The achievements in developing health professional regulation and regulatory capacity can help countries in the ECSA region meet national targets for HIV service delivery, universal health coverage, and a stronger health workforce. ARC provides an illustrative model for sustained change that is transferable to other regions and healthcare cadres with similar regulatory challenges.

## Additional files

Additional file 1: Regulatory Function Framework (PDF 598 kb)

Additional file 2: Regional ARC Summative Congresses and Learning Sessions, 2011–2016 (PDF 196 kb)

## Abbreviations

AIDS: Acquired Immunodeficiency Syndrome
ARC: African Health Professions Regulatory Collaborative
ART: Antiretroviral Therapy
ASPPH: Association of Schools and Programs of Public Health
CDC: U.S. Centers for Disease Control and Prevention
CPD: Continuing Professional Development
DENOSA: Democratic Nursing Organization of South Africa
ECSA: East Central and Southern Africa
ECSACON: East Central and Southern Africa College of Nursing
ECSA-HC: East Central and Southern Africa Health Community
HIV: Human Immunodeficiency Virus
NCSBN: National Council of State Boards of Nursing
NIMART: Nurse Initiated and Managed Antiretroviral Therapy
NIMTB: Nurse Initiated and Managed Tuberculosis
OSCE: Objective Structured Clinical Examination
PEPFAR: President’s Emergency Plan for AIDS Relief
RFF: Regulatory Function Framework
SOP: Scope of Practice
TA: Technical Assistance
TB: Tuberculosis
UNAIDS: Joint United Nations Programme on HIV/AIDS
WHO: World Health Organization

## Footnotes

1 The ARC Knowledge Gateway was created using the WHO’s free, web-based platform, allowing members to share a shared discussion board, virtual library and community calendar.

## References

[1] UNAIDS (2016) Global AIDS update. Available from: http://www.unaids.org/sites/default/files/media_asset/global-AIDS-update-2016_en.pdf. Joint United Nations Programme on HIV/AIDS. 12349391

[2] UNAIDS (2014) Fast-Track: Ending the AIDS epidemic by 2030. Available from: http://www.unaids.org/sites/default/files/media_asset/JC2686_WAD2014report_en.pdf. Joint United Nations Programme on HIV/AIDS.

[3] UNAIDS (2017) Ending AIDS: Progress towards the 90–90-90 Targets Available from: http://www.unaids.org/sites/default/files/media_asset/Global_AIDS_update_2017_en.pdf. Joint United Nations Program on HIV/AIDS.

[4] Long L, Brennan A, Fox MP, Ndibongo B, Jaffray I, et al. (2011) Treatment outcomes and cost-effectiveness of shifting management of stable ART patients to nurses in South Africa: an observational cohort. PLoS Med 8: e1001055. doi: 1001010.1001371/journal.pmed.1001055. Epub 1002011 Jul 1001019. 10.1371/journal.pmed.1001055 PMC3139666 21811402

[5] Church K, Machiyama K, Todd J, Njamwea B, Mwangome M (2017). Identifying gaps in HIV service delivery across the diagnosis-to-treatment cascade: findings from health facility surveys in six sub-Saharan countries. J Int AIDS Soc.

[6] Brennan AT, Long L, Maskew M, Sanne I, Jaffray I, et al. (2011) Outcomes of stable HIV-positive patients down-referred from a doctor-managed antiretroviral therapy clinic to a nurse-managed primary health clinic for monitoring and treatment. AIDS 25: 2027–2036. doi: 2010.1097/QAD.2020b2013e32834b36480. 10.1097/QAD.0b013e32834b6480 PMC3669640 21997488

[7] Martinez-Gonzalez NA, Tandjung R, Djalali S, Rosemann T (2015). The impact of physician-nurse task shifting in primary care on the course of disease: a systematic review. Hum Resour Health.

[8] Kredo T, Adeniyi FB, Bateganya M, Pienaar ED (2014) Task shifting from doctors to non-doctors for initiation and maintenance of antiretroviral therapy. Cochrane database Syst rev: CD007331. doi: 007310.001002/14651858.CD14007331.pub14651853. 10.1002/14651858.CD007331.pub3 PMC11214583 24980859

[9] Penazzato M, Davies MA, Apollo T, Negussie E, Ford N (2014) Task shifting for the delivery of pediatric antiretroviral treatment: a systematic review. J Acquir Immune Defic Syndr 65: 414–422. doi: 410.1097/QAI.0000000000000024. 10.1097/QAI.0000000000000024 24583614

[10] Iwu EN, Holzemer WL (2014) Task shifting of HIV management from doctors to nurses in Africa: clinical outcomes and evidence on nurse self-efficacy and job satisfaction. AIDS Care 26: 42–52. doi: 10.1080/09540121.09542013.09793278. Epub 09542013 May 09540123. 10.1080/09540121.2013.793278 23701374

[11] Mdege ND, Chindove S, Ali S (2013) The effectiveness and cost implications of task-shifting in the delivery of antiretroviral therapy to HIV-infected patients: a systematic review. Health Policy Plan 28: 223–236. doi: 210.1093/heapol/czs1058. Epub 2012 Jun 1026. 10.1093/heapol/czs058 22738755

[12] Nkhata MJ, Muzambi M, Ford D, Chan AK, Abongomera G (2016). Shifting human resources for health in the context of ART provision: qualitative and quantitative findings from the Lablite baseline study. BMC Health Serv Res.

[13] World Health Organization. Task shifting: rational redistribution of tasks among health workforce teams: global recommendations and guidelines: WHO; 2008. Available from: http://www.who.int/healthsystems/TTR-TaskShifting.pdf?ua=1

[14] Callaghan M, Ford N, Schneider H (2010). A systematic review of task- shifting for HIV treatment and care in Africa. Hum Resour Health.

[15] Lehmann U, Van Damme W, Barten F, Sanders D (2009) Task shifting: the answer to the human resources crisis in Africa? Hum Resour Health 7:49.: 10.1186/1478-4491-1187-1149. 10.1186/1478-4491-7-49 PMC2705665 19545398

[16] World Health Organization. Consolidated guidelines on the use of antiretroviral drugs for treating and preventing HIV infection recommendations for a public health approach: recommendations for a public health approach: WHO; 2016. Available from: http://apps.who.int/iris/bitstream/10665/208825/1/9789241549684_eng.pdf?ua=1 27466667

[17] McCarthy CF, Voss J, Verani AR, Vidot P, Salmon ME (2013). Nursing and midwifery regulation and HIV scale-up: establishing a baseline in east, central and southern Africa. J Int AIDS Soc.

[18] Hosey KN, Kalula A, Voss J (2016) Establishing an online continuing and professional development library for nurses and midwives in east, central, and Southern Africa J Assoc Nurses AIDS Care 27: 297–311. doi: 210.1016/j.jana.2016.1001.1007. Epub 2016 Jan 1029. 10.1016/j.jana.2016.01.007 PMC5407012 27086190

[19] Nkowane AM, Wheeler E (2014). Partnerships and collaboration: a means to effective regulation and practice for nursing and midwifery professions in the African region. Afr J Midwifery Womens Health.

[20] Zuber A, McCarthy CF, Verani AR, Msidi E, Johnson C (2014) A survey of nurse-initiated and -managed antiretroviral therapy (NIMART) in practice, education, policy, and regulation in east, central, and southern Africa. J Assoc Nurses AIDS Care 25: 520–531. doi: 510.1016/j.jana.2014.1002.1003. Epub 2014 Apr 1013. 10.1016/j.jana.2014.02.003 24739661

[21] Frenk J, Chen L, Bhutta ZA, Cohen J, Crisp N (2010). Health professionals for a new century: transforming education to strengthen health systems in an interdependent world. Lancet.

[22] McCarthy CF, Gross JM, Verani AR, Nkowane AM, Wheeler EL (2017). Cross-sectional description of nursing and midwifery pre-service education accreditation in east, central, and southern Africa in 2013. Hum Resour Health.

[23] Smith J, Odera DN, Chege D, Muigai EN, Patnaik P, et al. (2016) Identifying the gaps: an assessment of Nurses’ training, competency, and practice in HIV care and treatment in Kenya. J Assoc Nurses AIDS Care 27: 322–330. doi: 310.1016/j.jana.2016.1001.1005. Epub 2016 Jan 1025. 10.1016/j.jana.2016.01.005 27086191

[24] McCarthy CF, Voss J, Salmon ME, Gross JM, Kelley MA, et al. (2013) Nursing and midwifery regulatory reform in east, central, and southern Africa: a survey of key stakeholders. Hum Resour health 11:29.: 10.1186/1478-4491-1111-1129. 10.1186/1478-4491-11-29 PMC3706367 23800079

[25] McCarthy CF, Riley PL (2012). The african health profession regulatory collaborative for nurses and midwives. Hum Resour Health.

[26] Gross J, McCarthy C, Kelley M. Strengthening nursing and midwifery regulations and standards in Africa. Afr J Midwifery Womens Health. 2011;5

[27] McCarthy CF, Zuber A, Kelley MA, Verani AR, Riley PL. The African health profession regulatory collaborative (ARC) at two years. Afr J Midwifery Womens Health. 2014;8 10.12968/ajmw.2014.8.sup2.4 PMC4826056 27066113

[28] Gross JM, Kelley MA, McCarthy CF (2015). A model for advancing professional nursing regulation: the African health profession regulatory collaborative. Journal of Nursing Regulation.

[29] Chilomo C, Mondiwa M, Wasili R (2014). Strengthening professional development in Malawi. Afr J Midwifery Womens Health.

[30] Agricole W, Hoarau MA, D'Arc Suzzette J, Sinon E (2014). Review of the Seychelles’ nurses and midwives act. Afr J Midwifery Womens Health.

[31] (2003) The breakthrough series: IHI’s collaborative model for achieving breakthrough improvement. Boston: Institute for Healthcare Improvement.

[32] African Regulatory Collaborative (2013) Continuing professional development for nurses and midwives: a toolkit for developing a national CPD framework. ARC. Available from: http://www.africanregulatorycollaborative.com/Documents/ARCCPDToolkitApril2014_000.pdf.

[33] African Regulatory Collaborative (2014) ARC Legal Regulatory Matrix. Available from: http://www.africanregulatorycollaborative.com/ARC-Resources.html.

[34] McCarthy CF, Kelley MA, Verani AR, St Louis ME, Riley PL (2014). Development of a framework to measure health profession regulation strengthening. Eval Program Plann.

[35] Paulk MC, Weber, C. V., Curtis, B., & Chrissis, M. B., editor (1994) The capability maturity model: guidelines for improving the software process, reading. Massachusetts: Addison-Wesley.

[36] Dynes M, Tison L, Johnson C, Verani A, Zuber A (2016). Regulatory advances in 11 sub-Saharan countries in year 3 of the African health profession regulatory collaborative for nurses and midwives (ARC). J Assoc Nurses AIDS Care.

[37] Kelley MA, Spangler SA, Tison LI, Johnson CM, Callahan TL (2017). Promoting regulatory reform: the African health profession regulatory collaborative (ARC) for nursing and midwifery year 4 evaluation. J Nursing Regulation.

[38] Clarke D, Duke J, Wuliji T, Smith A, Phuong K (2016). Strengthening health professions regulation in Cambodia: a rapid assessment. Hum Resour health.

[39] World Health Organization (2016) Global strategy on human resources for health: workforce 2030. WHO. Available from: http://apps.who.int/iris/bitstream/10665/250368/1/9789241511131-eng.pdf?ua=1.

[40] Waters KP, Zuber A, Willy RM, Kiriinya RN, Waudo AN (2013). Kenya's health workforce information system: a model of impact on strategic human resources policy, planning and management. Int J Med Inform.

[41] Clarke D. Law, regulation and strategizing for health. Chapter 10. In: Schmets G, Rajan D, Kadandale S, editors. Strategizing national health in the 21st century: a handbook: WHO; 2016. Available from: http://apps.who.int/iris/bitstream/10665/250221/1/9789241549745-chapter10-eng.pdf?ua=1/.

[42] World Health Organization (2016) Global strategic directions for strengthening nursing and midwifery 2016–2020. WHO. Available from: www.who.int/hrh/nursing_midwifery/global-strategic-midwifery2016-2020.pdf. ISBN 978 92 4 151045 5. 29975030

[43] Middleton L, Howard AA, Dohrn J, Von Zinkernagel D, Parham Hopson D (2014). The nursing education partnership initiative (NEPI): innovations in nursing and midwifery education. Acad Med.

