# Supplementary material for: Evaluation of the impact of the ARC program on national nursing and midwifery regulations, leadership, and organizational capacity in East, Central, and Southern Africa
Source: BMC Health Serv Res. 2018 Jun 4;18:406. doi: 10.1186/s12913-018-3233-4 (PMC5987602; doi:10.1186/s12913-018-3233-4)
Supplement: Supplementary file 1 — Regulatory Function Framework (PDF 598 kb) [file 12913_2018_3233_MOESM1_ESM.pdf]

Additional File 1: Regulatory Function Framework

	STAGE 1	STAGE 2	STAGE 3	STAGE 4	STAGE 5
**Nursing and Midwifery Legislation**	☐ Identification of key issues with participation of stakeholders.<br><br>☐ Consensus around whether a new nursing and midwifery Act or amendments to existing legislation are needed.	☐ Legislation drafted with stakeholders including Ministry of Health, nursing and midwifery council and/or professional associations, academia, and legislature or parliament.	☐ Approval, commencement, and publication of legislation.	☐ Implementation through dissemination and training of nurses and midwives in their rights and duties.<br><br>☐ Issuance by Councils and/or Ministry of Health of rules or regulations.	☐ Monitoring and evaluation of compliance and impact.
**Registration System and Use of Registration Data**	☐ Registration is not legally required for nurses and midwives to practice<br>OR<br>Registration is lifelong (i.e. renewal is not required).<br><br>☐ The register is primarily a paper-based system.	☐ Renewal of registration (or license) is required.<br><br>☐ Both paper and electronic (e.g. Excel) system for registration is used.<br><br>☐ Registration system can answer basic queries (e.g. number of midwifes in the country).	☐ Registration system (including licensure and re-licensure) is primarily electronic (use of software).<br><br>☐ Database includes all public sector nurses and is regularly updated.<br><br>☐ Registration system can be queried to generate workforce reports.	☐ Registration system is completely electronic and includes all public and private sector nurses.<br><br>☐ Database displays various registration statuses of nurses and midwives.<br><br>☐ Database can be programmed to automatically generate workforce reports.	☐ Registration, licensure and re-licensure services are available online or are decentralized.<br><br>☐ Registration database can exchange data with other health information systems.<br><br>☐ Registration data used by decision makers for workforce policy and planning.

	STAGE 1	STAGE 2	STAGE 3	STAGE 4	STAGE 5
**Licensure Process**	☐ Licenses not required to practice	☐ Licenses are issued with initial registration (no separate licensure examination).<br><br>☐ Renewal of license is required at intervals specified by the regulatory authority.	☐ An examination or assessment process is in place for initial registration and licensure.<br><br>☐ The examination or assessment is paper-based.<br><br>☐ National competency standards are being developed.	☐ Examination or assessment content meets national competency standards.<br><br>☐ Various statuses of licenses issued (i.e. conditional, suspended).<br><br>☐ Licensure verification process facilitates entry of foreign educated nurses/midwives into workforce.	☐ Registration and initial licensure examination content is updated regularly.<br><br>☐ Examination content aligns with global guidelines or regional competency standards.<br><br>☐ The licensure status of a nurse or midwife is available to the public either via website, phone or in-person.
**Scope of Practice (SOP)**	☐ SOP not defined by legal statute or regulation.<br><br>☐ SOP may be decided by the employer or based on health facility needs.	☐ Council has the authority to formally define the SOP.<br><br>☐ SOP are under development.<br><br>☐ SOP reviewed or revised within 10 years.	☐ Nationally standardized SOP for all nurse and midwife categories.<br><br>☐ SOP is based on nursing/midwifery context, consultations and job descriptions.<br><br>☐ SOP reviewed or revised within last five years.	☐ SOP includes essential nursing/midwifery competencies.<br><br>☐ SOP is regularly and systematically reviewed and revised.<br><br>☐ SOP allows for individuals to make decisions about task shifting or task sharing.	☐ All SOP align with global guidelines and standards for nursing and midwifery.<br><br>☐ SOP reviewed and revised according to global standards.<br><br>☐ SOP is dynamic, flexible, and inclusive, not restrictive.
**Continuing Professional Development (CPD)**	☐ CPD does not exist.<br><br>☐ CPD is voluntary.<br><br>☐ CPD framework for nursing and midwifery may be in planning stages.	☐ Council has a mandate in legislation to require CPD.<br><br>☐ National CPD framework for nursing and midwifery is developed.<br><br>☐ Implementation of CPD requirement is in pilot or early stages.	☐ CPD program for nurses and midwives is finalized and nationally disseminated.<br><br>☐ CPD is officially required for re-licensure.<br><br>☐ Strategy in place to promote and track compliance.	☐ Electronic system in place to monitor CPD compliance.<br><br>☐ Penalties for non-compliance with CPD exist.<br><br>☐ Available CPD includes content on national HIV service delivery guidelines for nurses and midwives.	☐ Multiple types of CPD are available including web-based and mobile-based models.<br><br>☐ CPD content aligns with regional standards or global guidelines.<br><br>☐ Regular evaluations of CPD program carried out.

	STAGE 1	STAGE 2	STAGE 3	STAGE 4	STAGE 5
**Accreditation of Pre-Service Education**	☐ Council does not have legal authority to approve pre-service nursing/midwifery schools or programs.<br><br>☐ Public schools/programs may be "endorsed" by the council.	☐ Council has legal authority to approve pre-service schools/programs.<br><br>☐ Council issues standards for accreditation of nursing schools/programs.<br><br>☐ No time limit or expiration date on accreditation approval.	☐ Initial assessment visits are carried out by the council or their designated authority.<br><br>☐ Standards for accreditation are regularly reviewed and revised.<br><br>☐ Requirement for accreditation renewal is enforced.	☐ Assessment visits are regularly carried out by an independent body.<br><br>☐ Council has an electronic system to track accreditation status.<br><br>☐ Various levels of accreditation granted (i.e. probationary, conditional).	☐ Group independent from council makes accreditation determination for both public and private schools/programs.<br><br>☐ Accreditation standards align with global or regional guidelines.<br><br>☐ Accreditation status available to the public.
**Professional Misconduct and Disciplinary Powers**	☐ Council does not have authority to manage complaints and impose sanctions.<br><br>☐ Standards of professional conduct may not be defined.	☐ Legislation authorizes council to define standards for professional conduct.<br><br>☐ Council has authority to investigate or initiate inquiries into professional misconduct.<br>☐ Basic types of complaints and sanctions exist	☐ Complaints investigation and misconduct hearings are separate processes.<br><br>☐ A range of disciplinary measures (e.g. penalties, sanctions, conditions) exist.<br><br>☐ Appeals processes are available and accessible.	☐ The processes and documentation of complaints and sanctions are transparent.<br><br>☐ Processes and timelines are in place to review and remove penalties and sanctions.<br><br>☐ Processes are in place for members of the public to lodge a complaint.	☐ Professional conduct standards align with regional standards or global guidelines.<br><br>☐ The complaint management process is regularly evaluated for transparency and timeliness.<br><br>☐ Information on complaints and sanctions is available to the public.

Additional File 1, the RFF, is reprinted from *Evaluation and Program Planning*, 46, McCarthy, C. F., Kelley, M. A., Verani, A. R., St Louis, M. E., & Riley, P.L. "Development of a framework to measure health profession regulation strengthening," p. 20. doi:10.1016/j.evalprogplan.2014.04.008 (2014), with permission from Elsevier: https://www.sciencedirect.com/science/article/pii/S0149718914000470.
