# Supplementary material for: Evaluation of the impact of the ARC program on national nursing and midwifery regulations, leadership, and organizational capacity in East, Central, and Southern Africa
Source: BMC Health Serv Res. 2018 Jun 4;18:406. doi: 10.1186/s12913-018-3233-4 (PMC5987602; doi:10.1186/s12913-018-3233-4)
Supplement: Supplementary file 2 — Regional ARC Summative Congresses and Learning Sessions, 2011–2016 (PDF 196 kb) [file 12913_2018_3233_MOESM2_ESM.pdf]

Additional File 2: Regional ARC Summative Congresses and Learning Sessions, 2011-2016 [44]

	SUMMATIVE CONGRESS	1ST LEARNING SESSION	2ND LEARNING SESSION
**YEAR 1**	Nairobi, Kenya<br>28 February to 2 March 2011	Durban, South Africa<br>24-26 June 2011	Arusha, Tanzania<br>5-7 October 2011
**YEAR 2**	Johannesburg, South Africa<br>20-22 June 2012	Pretoria, South Africa<br>18-20 September 2012	Gaborone, Botswana<br>6-8 February 2013
**YEAR 3**	Nairobi, Kenya<br>30 July to 2 August 2013	Nairobi, Kenya<br>4-6 February 2014	Lusaka, Zambia<br>29 April to 1 May 2014
**YEAR 4**	Windhoek, Namibia<br>24-26 February 2015	Johannesburg, South Africa<br>14-16 July 2015	Harare, Zimbabwe<br>10-12 November 2015
**YEAR 5**	Johannesburg, South Africa<br>16-18 February 2016		
